# *Haematococcus pluvialis* as a Potential Source of Astaxanthin with Diverse Applications in Industrial Sectors: Current Research and Future Directions

**DOI:** 10.3390/molecules26216470

**Published:** 2021-10-27

**Authors:** Siti Nur Hazwani Oslan, Joo Shun Tan, Siti Nurbaya Oslan, Patricia Matanjun, Ruzaidi Azli Mohd Mokhtar, Rossita Shapawi, Nurul Huda

**Affiliations:** 1 Faculty of Food Science and Nutrition, Universiti Malaysia Sabah, Kota Kinabalu 88400, Sabah, Malaysia; snhazwanioslan@ums.edu.my (S.N.H.O.); patsy@ums.edu.my (P.M.); 2 Bioprocess Technology, School of Industrial Technology, Universiti Sains Malaysia, Gelugor 11800, Pulau Pinang, Malaysia; jooshun@usm.my; 3 Department of Biochemistry, Faculty of Biotechnology and Biomolecular Sciences, Universiti Putra Malaysia, Serdang 43400, Selangor, Malaysia; snurbayaoslan@upm.edu.my; 4 Biotechnology Research Institute, Universiti Malaysia Sabah, Kota Kinabalu 88400, Sabah, Malaysia; ruzaidi@ums.edu.my; 5 Borneo Marine Research Institute, Universiti Malaysia Sabah, Kota Kinabalu 88400, Sabah, Malaysia; rossita@ums.edu.my

**Keywords:** *Haematococcus pluvialis*, bioactive compounds, astaxanthin, antioxidant bioactivity, health benefits

## Abstract

*Haematococcus pluvialis*, a green microalga, appears to be a rich source of valuable bioactive compounds, such as astaxanthin, carotenoids, proteins, lutein, and fatty acids (FAs). Astaxanthin has a variety of health benefits and is used in the nutraceutical and pharmaceutical industries. Astaxanthin, for example, preserves the redox state and functional integrity of mitochondria and shows advantages despite a low dietary intake. Because of its antioxidant capacity, astaxanthin has recently piqued the interest of researchers due to its potential pharmacological effects, which include anti-diabetic, anti-inflammatory, and antioxidant activities, as well as neuro-, cardiovascular-, ocular, and skin-protective properties. Astaxanthin is a popular nutritional ingredient and a significant component in animal and aquaculture feed. Extensive studies over the last two decades have established the mechanism by which persistent oxidative stress leads to chronic inflammation, which then mediates the majority of serious diseases. This mini-review provides an overview of contemporary research that makes use of the astaxanthin pigment. This mini-review provides insight into the potential of *H. pluvialis* as a potent antioxidant in the industry, as well as the broad range of applications for astaxanthin molecules as a potent antioxidant in the industrial sector.

## 1. Introduction

*Haematococcus pluvialis* is a unicellular freshwater microalga that is a promising source of bioactive substances, such as carotenoids, proteins, and fatty acids (FAs), particularly astaxanthin, a powerful antioxidant [1]. Even though there are various organisms capable of producing astaxanthin, only a small number of these organisms are commercially cultivated. Among the commercially potential microalgae, *H. pluvialis* is known to be one of the richest sources of natural astaxanthin as it can accumulate large quantities of astaxanthin compared to other organisms [2]. Astaxanthin, also known as 3,3′-dihydroxy-ß-carotene-4,4′-dione, is a secondary carotenoid with a brilliant blood-red colour that can be directly generated by applying cellular stressors to *H. pluvialis* [3]. During the life cycle of *H. pluvialis* cells, ultra-structural alterations occur when the cells transition from green to red. The chemical composition of the cellular substance evolves as well. Based on dry biomass weight (DBW), during the “green phase,” up to 1% lutein and total lipid content varies from 20–25, whereas the “red phase” content 32–37% of lipids and deposit 1–5% of astaxanthin [4].

Compounds with a high value-added, such as astaxanthin, lutein, and ß-carotene, as well as fatty acids, provide numerous health benefits. Astaxanthin and lutein are two carotenoids that are well known as natural antioxidants. Astaxanthin is one of the most potent carotenoid compounds on the market. Carotenoids have piqued the interest of researchers in recent decades due to their powerful antioxidant, mending, antiproliferative, anti-inflammatory, and possibly antiaging properties. They can be utilised to prevent diseases caused by oxidative stress and chronic inflammation [5]. Astaxanthin has 500 times the antioxidant activity of vitamin E and a 38-fold greater potential to terminate free radical chain reactions than ß-carotene [4]. Furthermore, astaxanthin is an anti-inflammatory, having therapeutic effects on a variety of human ailments such as UV-light photo-oxidation, inflammation, cancer, liver function, and heart, joint, skin, ageing, and prostate health [1,6]. In addition, lutein is a pigment found in the macula of the eye. Its presence in ocular tissue maintains the health of the eyes and may lessen the likelihood of age-related macular degeneration [7]. Natural astaxanthin from *H. pluvialis*, for example, has been shown to be safe as a supplement with no adverse effects for human health and as a feed additive in the aquaculture and animal feed industries [4,8], and no reported negative effects have been identified over its 20 years of usage as a dietary supplement [9]. The European Commission Implementing Regulation (EU) 2017/2470 states that astaxanthin-rich oleoresin produced from *H. pluvialis* can be absorbed at rates of up to 40–80 mg/day in dietary supplements [1]. The total carotenoids intake, however, should be in the 5–10 mg/day range. This includes a safe level of ß-carotene exposure of less than 15 mg/day [10].

Researchers are constantly confirming the beneficial effects of astaxanthin in the treatment of human and animal diseases based on its potential in pharmacological effects, such as anti-diabetic, anti-inflammatory, food additive, and antioxidant activities, as well as cardiovascular, ocular, and skin-protective effects [5]. In this mini-review, a special emphasis on compounds of interest of *H. pluvialis* is presented on the wide variety of applications of astaxanthin as a protective antioxidant in the industry.

## 2. Biochemical Composition of *Haematococcus pluvialis*

### 2.1. Proteins and Carbohydrates

Cell maturation and progression through successive stages of the life cycle result in a change in the cell’s biochemical profile. Most *H. pluvialis* strains have a protein content of 29–45% per dry weight in the green stage, under favourable growth conditions [3]. However, protein content in the palmella stage of the life cycle could be reduced to 36%. Furthermore, during the red stage of *H. pluvialis* cultivation, protein was estimated to contribute 21–23% of the cellular content [2]. The amino acid composition of proteins in the red stage revealed that proteins were primarily composed of aspartic acid, glutamic acid, alanine, and leucine, with a total amino acid content of 10.02/100 mg, 46% of which belonged to essential amino acids [11]. Carbohydrate content in the green stage is approximately 15–17% [2]. Carbohydrates accumulate in higher concentrations in *H. pluvialis* during the red stage when exposed to stress conditions, such as high acidity, nutrient deficiency, light stress, or temperature variations. It was estimated that it could rise by up to 63% on the first day of stress exposure [12]. Furthermore, under prolonged stress conditions, *H. pluvialis* cells consumed starch carbohydrate [3]. 

### 2.2. Lipid

Total lipid content ranges from 20 to 25% in the green stage, with approximately 10% lipids retained in the chloroplasts consisting primarily of polyunsaturated fatty acids (PUFAs) [3]. Red stage cells can generate up to 40% of their cell weight in the form of cytoplasmic lipid droplets (LD) and a significant amount of secondary metabolites, including up to 4% of the ketocarotenoid astaxanthin. The total fatty acid profile of *H. pluvialis* is relatively flexible and can change depending on strain, including palmitic, linoleic, and linolenic acids [2]. This variation is attributed to a variety of factors, including stress conditions caused by limiting nitrogen and phosphorus content, culture environment, culture variations, and strain origin [4]. Moreover, the higher lipid content of *H. pluvialis* grown under nutrient depletion [13]. According to many studies, using unfavourable conditions resulted in a significant increase in total lipid when compared with control group culture. 

### 2.3. Carotenoid

The carotenoid fraction of green vegetative cells is primarily composed of lutein (75–80%), ß-carotene (10–20%), and others, including chlorophyll a and b, primary carotenoids, zeaxanthin, canthaxanthin, echinenone, violaxanthin, neoxanthin, and lactucaxanthin [2]. The total carotenoid content is significantly increased in the red stage, with primary carotenoids of the green stage pattern being replaced by secondary carotenoids, astaxanthin (80–99% of total carotenoids) [14]. The *H. pluvialis*-derived astaxanthin has a monoester-to-diester ratio of roughly 70%, a diester-to-free form ratio of 25%, and a free form ratio of 5%. The *H. pluvialis* contains a variety of fatty acids that are stored in the form of cytoplasmic lipid droplets as triacylglycerol (TAG) [15]. Under certain stress conditions, the *H. pluvialis* has been shown to accumulate up to 3–5% DW of astaxanthin [4]. Furthermore, in carotenoids oleoresin extracted from *H. pluvialis* by supercritical CO_2_ treatment is high [16]. The antioxidant content of the oleoresin is important for food applications. It has the potential to be a highly effective antioxidant, balancing oxidative and inflammatory status. Ruiz-Dominguez et al. (2019) [16] discovered that the oleoresin of *H. pluvialis* contained 96.22 mg/g of total astaxanthin (including free astaxanthin and astaxanthin esters) and was mostly composed of unsaturated fatty acids (78% of total fatty acids).

Following harvest from cultivation systems, the cell walls of microalgae, due to their robustness, likely represent the most significant barrier to target compound extraction. In recent years, several different methods for extracting astaxanthin from biomass have been reported for industrial microalgae, including homogenization, ultrasonication, microwave, solvent, acid, edible oils, and supercritical CO_2_ (SC-CO_2_) [2]. These methods aim to maximise the extraction efficiencies of target bio-products. Recently, Alvarez et al. (2020) [17] used supercritical CO_2_ extraction to determine the best operating parameters for recovering astaxanthin and fatty acids from *H. pluvialis*. The results showed that at 50 °C/50 MPa, astaxanthin recoveries of 95% were possible after 175 min for a CO_2_ flow rate of 2 L/min and 95 min for a CO_2_ flow rate of 4 L/min. Furthermore, whereas CO_2_ has a minimal critical temperature, supercritical CO_2_ extraction can be carried out at low temperatures, preventing astaxanthin degradation. Furthermore, CO_2_ is relatively inexpensive, inert, widely accepted as safe, and non-toxic [2].

## 3. Interaction Astaxanthin and Reactive Oxygen Species (ROS)

Reactive oxygen species (ROS) are produced in the cell by a variety of enzymes, including the cytoplasmic membrane NADPH oxidase; the mitochondrial respiratory chain enzyme complex; endoplasmic reticulum, peroxisome, and other enzymes, such as xanthine oxidase, lipo- and cyclooxygenase, and cytochromes P450 [18]. Mitochondria are critical for maintaining cellular redox equilibrium; hence, preserving their structural and functional integrity is crucial for efficient cellular function [5]. According to Landon et al. (2020) [19], astaxanthin’s bioactivity increases mitochondrial function by lowering mitochondrial reactive oxygen species (mtROS) generation while improving ATP production. An imbalance between prooxidants and antioxidants causes oxidative stress, which causes macromolecular damage and disrupts redox signalling and cellular regulation [20]. Prooxidants are substances that aid in the production of ROS, which then degrade biological macromolecules such as DNA [21]. Increased ROS production can damage biological structures and has been linked to a number of chronic conditions [18]. Antioxidants help to minimise oxidative stress by counteracting or reducing the effects of ROS [19].

Several research papers on the interaction between astaxanthin and ROS have been published [2,18]. The development of acute and chronic disorders is heavily influenced by the harmful effects of reactive species. This is because free radicals prefer to attack nucleic acids (RNA and DNA) and proteins [22]. Furthermore, astaxanthin has been utilised as a targeted drug to scavenge free radicals at specific sites via a carrier as a strong antioxidant to protect cells injured by oxidation [20]. Inflammation is associated to the aetiology of cardiovascular disease, neurological illnesses, and ageing, as are high levels of prooxidants and different markers of oxidative stress, as well as cell and tissue damage [23]. Antioxidants can minimise or prevent oxidation of oxidised substrates and quickly absorbed ions, remove free radicals, and chelate redox metals at physiologically relevant amounts when added to a cell [24]. Free radicals, in particular, have one or more unpaired electrons, making them reactive and capable of triggering chain reactions via propagating molecular damage. For instance, ROS are the source of the majority of free radicals [18].

As a result, the primary function of astaxanthin as an antioxidant is to deactivate reactive oxidants has been reported [25]. The oxygen depletion, quenching of singlet oxygen molecules, scavenging of ROS or termination of a chain reaction of oxidation propagation, chelation of metal ions that could otherwise catalyse ROS formation, and repair of oxidative damage are all processes that antioxidants have been involved in to protect a biological system against oxidative damage [26].

## 4. Potential Application of Astaxanthin in Industry

Basis of current benefits, astaxanthin has prospective application value in human therapy, such as anti-diabetic, anti-inflammatory, and anti-aging properties, and it is advantageous in the food and feed aquaculture sector.

### 4.1. Anti-Diabetic

Natural astaxanthin administration has not been linked to any negative side effects in trials [27]. As a result, astaxanthin has been explored in depth as an anti-diabetic agent in anti-diabetic drugs. Zhuge et al. (2020) [8] discovered that astaxanthin is a strong antioxidant that can control and prevent diet-induced insulin resistance and hepatic steatosis in rats. To investigate the anti-diabetic benefits of astaxanthin, male Wistar rats were fed a high-energy diet containing astaxanthin in the range of (15 to 50 mg/kg), followed by a low dose streptozotocin (STZ) (40 mg/kg) injected to generate the diabetes model. The results reveal that astaxanthin administration has a significant effect in vivo by increasing expression of insulin sensitivity associated genes (adiponectin, adipoR1, and adipoR2), consequently attenuating STZ-induced diabetes. For example, Vega et al. (2015) [28] found that giving rats significant dosages of ASX (up to 1240 mg/kg/day) for a lengthy period of time (90 days) had no harmful effect. Another study by Arunkumar et al. (2012) found that 6 mg/kg per day for 45 days significantly reduced plasma glucose and insulin levels in HFFD (high fat fructose diet)-fed rats and enhanced insulin sensitivity [29]. Furthermore, astaxanthin at doses ranging from 8 to 45 mg/day for 4 to 12 weeks has little side effects in people [30]. Furthermore, Landon et al. (2020) [19] evaluated the mechanism of astaxanthin has been explored on type 1 and type 2 DM animal models, administered orally or parenterally. The astaxanthin decreases insulin resistance and secretion, lowers hyperglycaemia, and protects against retinopathy, nephropathy, and neuropathy. Furthermore, in a randomised, placebo-controlled clinical investigation, astaxanthin was found to be promising in improving glucose and lipid metabolism. Mashhadi et al. (2018) [31] observed that astaxanthin (8 mg/day for 8 weeks) supplementation significantly raised serum adiponectin (4714 g/mL) compared to placebo and baseline (4513 and 3615 g/mL, respectively).

### 4.2. Anti-Inflammatory Properties

Several research papers have shown that astaxanthin supplemented with diet has a high antioxidant activity in mice [32,33,34]. In biological systems, astaxanthin can stop the induction of inflammation. Astaxanthin, a natural strong antioxidant xanthophyll carotenoid, has been demonstrated to contain anti-inflammatory activity, and it has been reported that it has protective benefits against acute lung injury [32] due to its great antioxidant capabilities. Astaxanthin protects against LPS-induced lung damage in vitro and in vivo by inhibiting the TLR4/MyD88 signalling pathway. In addition, astaxanthin can also prevent lipopolysaccharide-induced acute lung damage and sepsis in mice via the MAPK/NF-B signalling pathway. Astaxanthin, for example, can be utilised to protect the heart by inhibiting oxidative stress and apoptosis in rat coronary artery microembolism (CME)-caused cardiac chambers [33] and decreasing exercise-related damage to the skeletal muscles and heart in mice [35]. Furthermore, astaxanthin has been found to play a role in cardioprotection as well as the prevention and treatment of ochratoxin A (OTA)-produced heart damage in mice [34]. Astaxanthin is an antioxidant that modulates redox balance and is thought to be a significant regulator of inflammatory responses in cardiovascular disorders. In addition to its antioxidant properties, which are advantageous to the cardiovascular system, it helps to prevent diseases, such as atherosclerosis, arterial hypertension, and dyslipidemia [36]. Astaxanthin has been shown in in vitro studies to prolong LDL oxidation in a dose-dependent way [37]. In patients with diabetes mellitus, LDL oxidation is also linked to the development of endothelial dysfunction, which raises the risk of cardiovascular problems [38]. Furthermore, it is more effective than lutein and tocopherol. In turn, blood samples from individuals who were supplemented daily were analysed, and the best benefit was found at a dose of 14.4 mg astaxanthin for 14 days, there was a considerable delay in LDL oxidation [37].

### 4.3. The Food Industry

Recent functional food ingredient research has revealed a significant interest in the production of foods containing green algal elements, particularly astaxanthin. Astaxanthin is also frequently utilised as a dietary supplement in the food and feed industries. The EFSA Panel on Nutrition, Novel Foods, and Food Allergens concluded in 2020 that an adult intake of 8 mg of astaxanthin from food supplements is safe [39]. Furthermore, oxidation is one of the key reasons of food quality deterioration for meat products in the food sector. Manufacturers of sausages commonly utilise synthetic antioxidants, such as butylated hydroxytoluene (BHT), to prevent oxidation without reducing shelf life and nutritional loss, respectively. Synthetic antioxidants, on the other hand, while helpful in the oxidation process, are a possible source of carcinogens. The substitution of natural astaxanthin resulted in higher oxidation stability in sausages, and the level of prevention of malondialdehyde formation on storage days was comparable to that of butylated hydroxytoluene (BHT) [40].

Recent research has shown that adding microalgae powder or extract to food products, such as cookies, can improve the nutritional and textural aspects [41,42,43]. Sahni et al. (2019) [43] investigated the use of chlorophyll from *Chlorella* sp. (Abca-17) as an ingredient in the making of cookies and replaced wheat flour, resulting in texture changes with the addition of 1–12% of the microalgal meal, i.e., the weight and thickness increased, but the diameter, spread ratio, and spread factor dropped with increasing content. However, adding *Arthrospira platensis* powder to a regular cookie recipe at quantities of up to 3% resulted in increased hardness and lower sensory scores [42].

### 4.4. Aquaculture and Broiler Industry

Aside from its antioxidant capacity, astaxanthin can also be employed as a diet addition in aquaculture to increase growth performance [44,45] and in the broiler business [46,47]. Currently, Xie et al. (2020) [45] investigated that administering astaxanthin (75 and 150 mg/kg) in a high fat diet of juvenile largemouth bass (*Micropterus salmoides*) in aquaculture nutrition affects growth performance, lipid metabolism, and immunological response. After supplementing fish with astaxanthin, oxidative stress was reduced, superoxide dismutase activity enhanced, and malondialdehyde level decreased. Furthermore, increasing dosages of dietary astaxanthin (50 to 150 mg/kg) significantly lowered blood cholesterol and triglyceride levels in Asian seabass, *Lates calcarifer* [44]. According to Lim et al. (2019) [42], this study found that when fish were fed varied diets containing high levels of astaxanthin, their haematological and indices (white blood cell count, red blood cell count, haemoglobin, and haematocrit) improved significantly.

Furthermore, this carotenoid pigment is best recognised as an important aquacultural feed additive for giving the pinkish-red colouration to the flesh of salmons, trouts, ornamental fish, shrimp, lobsters, and crabs, resulting in improved quality and customer acceptance. According to Lim et al. (2017) [48], after a single oral dose of 500 lg of astaxanthin supplementation in rainbow trout, the plasma astaxanthin concentration was considerably enhanced after 24 h. Meanwhile, a single dose of 100 lg astaxanthin resulted in a maximal concentration of astaxanthin in rainbow trout blood after 18 h.

Additionally, Tzanova et al. (2017) [49] reported that using astaxanthin and canthaxanthin as dietary supplements in fish farms can enhance the organoleptic qualities of salmonid products and help prevent reproductive diseases. They discovered that a loss of egg colour, which indicates a deficient astaxanthin content, is proportional to offspring mortality. Moreover, the colour of salmonid eggs is determined by the presence of carotenoid pigments, primarily astaxanthin and canthaxanthin. According to Atanasov et al. (2015) [50], orange eggs from three trout species cultured in Bulgaria—rainbow trout (*Oncorhynchus mykiss)*, salmon trout (*Salmo trutta m. fario L*.), and brook trout (*Salvelinus fontinalis M.*—had higher fertility and a lower offspring mortality rate than light yellow eggs. Moreover, the experiments were conducted on salmon diagnosed with the M74 syndrome. They hypothesised that nutrient prophylaxis during the feeding period of male and female fish could help prevent high fry mortality. Thus, determining the concentrations of astaxanthin and canthaxanthin in fish eggs may aid in the early detection and prevention of M74 syndrome. A minimum astaxanthin and canthaxanthin content of 2.22 mg/kg in eggs has been reported to be effective in preventing or control the M74 syndrome [49].

Antioxidants have lately gained popularity in the poultry sector strongly dependent on oxidative stress and meat quality. Currently, Hosseindoust et al., (2020) [46] revealed that supplementing broiler chicks with astaxanthin (40 or 80 mg/kg) is an effective strategy to improve the level of total carotenoid in the liver, breast, and thigh of broiler chickens. Furthermore, the study found that increasing the amount of astaxanthin in the diet increased the activity of superoxide dismutase (SOD), while decreasing the amount of malondialdehyde (MDA) in the meat and increasing the redness or yellowness of the meat in broiler chickens [51]. For instance, it was confirmed that as the amounts of astaxanthin in the diet grew, the MDA concentration in meat decreased. Astaxanthin antioxidant activity is mostly related with interactions with active radicals such as hydroxyl and peroxyl radicals. It was performed on Pekin ducks, and the growth performance and meat quality have both improved as the discoloration or yellowish colour of the meat has increased. Dansou et al. (2021) [47] recently discovered that even high levels of astaxanthin (213.4 mg/kg) from *H. pluvialis* in the diet have no negative effect on laying hen process performance. However, supplementing astaxanthin at modest doses (21.3 and 42.6 mg/kg) promotes satisfactory egg fortification and laying hen health. It has been demonstrated that astaxanthin contains antioxidant and anti-inflammatory characteristics, which contribute to the improvement of animal health during administration.

### 4.5. Anti-Ageing

Astaxanthin is a carotenoid that has strong antioxidant and anti-inflammatory properties. Age-related skin changes are assumed to be caused by two primary mechanisms: biological ageing and UV light exposure (photoaging). Photoaging causes the destruction of extracellular matrix components (collagen and elastin), resulting in wrinkles, pigmentation, and degradation of skin texture [6]. Furthermore, UV light causes the skin to produce reactive oxygen species [52]. Through its anti-inflammatory impact, astaxanthin supplementation may prevent age-related skin degradation and preserve skin problems related to the environmental damage.

For example, Tominaga et al. (20A17) [53] found that a 6 mg or 12 mg dose of astaxanthin or a placebo reduced UVB-induced inflammatory cytokine release in keratinocytes and interleukin-1 levels in the stratum corneum significantly increased in the placebo and low-dose groups but not in the high-dose group between weeks 0 and 16 in healthy females. Thus, decreasing oxidative stress by suppressing inflammatory cytokines is critical for preventing age-related skin degeneration. Furthermore, Chalyk et al. (2017) [52] observed that the levels of malondialdehyde (MDA), a recognised indicator of systemic oxidative stress, were reduced after a human model received 4 mg daily doses of astaxanthin (by 11.2% on day 15 and by 21.7% on day 29). Furthermore, Seong-Ryeong et al., (2021) [54] revealed astaxanthin as a nutritional supplement that prevents the skin fibroblastic autophagic cell death generated by bisphenol A (BPA) in normal human dermal fibroblasts (NHDF), as well as reduced the phosphorylation of extracellular signal-regulated kinases (ERK) driven by ROS generation. In NHDF, BPA dramatically increased apoptotic cell death and autophagy. The reduction of intracellular ROS production by astaxanthin (10 M) effectively restored autophagic cell death induced by BPA.

Table 1 summarises the current state of research on astaxanthin and its role over the years.

## 5. Future Direction on Astaxanthin

Countries with a big coastline area (like Malaysia, Indonesia, and Australia) that are rich in biodiversity, especially microalgae, can be used for the isolation of new microalgae strain *H. pluvialis* as the sources of natural astaxanthin. As Malaysia has great potential to develop its abundant natural resources in order to increase the market for microalgae products. It probably will coordinate and provide a holistic effort among scientists, manufacturers, traders, health care professionals, and regulatory authorities to drive the industry to meet consumer expectations of quality, safety, and efficacy in microalgae products. Meanwhile, effective uses for astaxanthin extraction employing *H. pluvialis* biomass have yet to be established. If the mechanisms are completely understood, the proposed effort will seek to provide generic guidelines for astaxanthin productivity and greater yields of active chemicals for many applications. These requirements force many great application’s from astaxanthin, such as food producers, to look for new added functional ingredients with high nutritional content and bio activity that are sourced from natural sources in a sustainable manner and may give health advantages in addition to the traditional nutrients they contain. The addition of astaxanthin in applications increased many performances to the point where it was alone.

## 6. Conclusions

In conclusion, the green microalga *H. pluvialis* is the most appealing natural source for astaxanthin production. Astaxanthin is more antioxidant than ß-carotene, lycopene, and lutein. Many research institutions and food producers are interested in astaxanthin because of its antioxidant, anti-inflammatory, anti-diabetic, and anti-aging properties. The pharmacological effects of astaxanthin have been studied extensively in both humans and animals. Moreover, ROS-induced cellular imbalance caused macromolecular damage and is linked to many diseases. The biological significance of astaxanthin has been studied in vitro and in vivo. However, more research on astaxanthin is needed. Some experiments must be validated before the actual processes underlying astaxanthin effects can be described.

## Figures and Tables

**Table 1:** Summarised recent research associated with astaxanthin consumption in different application.

Model	Application	Remarks	References
Human	Anti-ageing	After 16 weeks of treatment with an oral (6 or 12 mg) dose of astaxanthin on healthy females, wrinkle metrics and skin moisture content dramatically worsened in the placebo group. In reaction to UVB irradiation, astaxanthin reduced inflammatory cytokine release from epidermal keratinocytes and MMP-1 secretion from dermal fibroblasts.	[53]
Human	Anti-ageing	Malondialdehyde (MDA) levels in a human model were reduced after treatment with 4 mg dosages daily (by 11.2% on day 15 and 21.7% on day 29).	[52]
Human	Skin health	Astaxanthin, a nutritional supplement that inhibits the phosphorylation of extracellular signal-regulated kinases (ERK) stimulated by ROS generation, was found to suppress the skin fibroblastic autophagic cell death produced by bisphenol A (BPA) in normal human dermal fibroblasts (NHDF).	[54]
Mice	Lung injury	In a mouse model of LPS-induced sepsis and acute lung injury, astaxanthin greatly increased survival and prevented lung injury.	[32]
Mice	Cardiac injury	The mechanism of ASX on the myocardium involves the Keap1-Nrf2 signalling system and the mitochondria-mediated apoptosis pathway, and it offers a mechanistic justification for the process leading OTA-induced myocardial injury. In mice, astaxanthin improved heart rate, cardiac enzymes, and antioxidant levels.	[34]
Human	Anti-diabetic	Astaxanthin has been shown to improve glucose metabolism and lower blood pressure in patients with type 2 diabetes mellitus.	[31]
Rat	Anti-diabetic	In STZ-induced diabetic rats, astaxanthin dramatically reduced blood glucose and total cholesterol (TC) levels while increasing blood levels of high-density lipoprotein cholesterol (HDL-C) in a dose-dependent way.	[8]
Cookies	Food	When the astaxanthin powder was increased up to 15% in the formulation, in vitro digestion Glycaemic Glucose Equivalent (GGE analysis) of the cookies revealed significantly lower glucose release, and the combination of astaxanthin with wholemeal flour significantly improved the antioxidant properties in the cookies.	[41]
Sausages	Food	The influence of variations in Peroxide value (PV), 2-thiobarbituric acid-reactive substances (TBARS), thiol content, textural profile analysis, instrumental colour, and sensory qualities on certain storage days has been documented with replacement with natural astaxanthin.	[40]
Laying hens	Broiler	In laying hens, the activity of superoxide dismutase (SOD) and glutathione peroxidase (GSH-Px) in serum were comparable at moderate doses (42.6 mg/kg) and high doses (213.4 mg/kg). Astaxanthin increased SOD and GSH-Px activity while also lowering MDA levels in the liver and serum.	[47]
Broiler chicken	Broiler	In the livers of broiler chickens, supplementation with 40 or 80 mg/kg astaxanthin dramatically reduced heat shock protein (HSP) 27, HSP70, tumour necrosis factor alpha, and interleukin-6 expression. In broilers under acute stress, the therapy reduced hyperthermic stress, enhanced meat quality, and increased antioxidant stability.	[46]
Fish (*Micropterus salmoides*)	Aquaculture	Dietary astaxanthin supplementation (75 and 150 mg/kg) increased the growth of juvenile largemouth bass fed a high-fat diet (HFD), as well as the hepatosomatic index and intraperitoneal fat ratio. Astaxanthin addition in the HFD reduced fish oxidative stress.	[45]
Fish Asian seabass, (*Lates calcarifer*)	Aquaculture	Fish immunological measures (respiratory burst activity, lysozyme activity, phagocytic activity, and serum total immunoglobulin) were significantly stimulated (*p* < 0.05) after a 90-day dietary intervention with astaxanthin ranging from 50 to 150 mg/kg.	[44]

## Data Availability

Not applicable.
